# Epidemiology of Injuries Seen in a Nigerian Tertiary Hospital

**DOI:** 10.4103/njcp.njcp_263_17

**Published:** 2018-06

**Authors:** NO Onyemaechi, OE Nwankwo, RA Ezeadawi

**Affiliations:** Department of Surgery, College of Medicine, University of Nigeria, Ituku–Ozalla,; 1 Department of Surgery, Enugu State University, Teaching Hospital, Parklane, Enugu, Nigeria

**Keywords:** Epidemiology, injuries, Nigeria, pattern, road traffic accidents

## Abstract

**Background:**

The study aimed to describe the pattern of injuries among patients presenting at a tertiary care hospital in Enugu southeast Nigeria.

**Patients and Methods:**

A retrospective review of records of all injured patients seen in our hospital over a 12–month period was done.

**Results:**

A total of 789 patients had complete medical records and were included in the study. Road traffic accident (RTA) was the most common cause of injury. Lacerations/abrasions, fractures, and traumatic brain injury (TBI) were the most frequently seen injuries. The injury severity score (ISS) of the patients ranged from 1 to 50 with a mean score of 8.9 ± 3.5. RTAs were responsible for 90.8% of patients with ISS >15. Patients with ISS >15 contributed to 64.1% of all deaths. The mortality rate was 4.5%. Most deaths resulted from RTA and were associated with TBI (*P* = 0.001).

**Conclusion:**

Lacerations and fractures were the most common injuries. RTA was the leading cause of injury. TBI was the most common cause of injury–related death.

## Introduction

Injuries are a major problem in both developing and developed countries. Trauma is a neglected public health problem in developing countries with over 90% of the world’s injury deaths occurring in low- and middle-income countries.^[1]^ Injuries represent 12% of the global disease burden and the third most common cause of death globally.^[2]^ Up to 90% of the global injury burden is borne by developing countries as a result of industrialization, motorized transportation, and armed ethno-religious conflicts.^[3,4]^

In Nigeria, reports show that trauma is the main reason for emergency room admission.^[5]^ Road traffic injuries (RTI) are the most frequently encountered injuries and the leading cause of death from trauma in Nigeria.^[6,7]^ However, the pattern and etiology of injuries vary among regions and countries. The use of motorcycles and tricycles as means of commercial transportation in parts of Nigeria may change the type and prevalence of injuries from road traffic accidents (RTAs). The recent surge in the spate of armed militancy and terrorism in some parts of Nigeria may also affect the prevalence and outcome of firearm–related injuries.

Many studies^[5–7]^ have described various aspects of injuries in different parts of Nigeria; however, the pattern of injuries in our subregion has been underreported. Understanding the epidemiology of injuries helps to analyze risk factors, develop effective preventive measures, and reduce morbidity and mortality from injuries. In many parts of Nigeria, trauma registries are nonexistent; documentation of injuries is inadequate, thus making trauma research problematic. Furthermore, paucity of injury epidemiology data limits the development of effective trauma management systems.^[4,8]^

The aim of this study was to describe the pattern and characteristics of injuries in our environment which is important for the development of effective trauma management system in our subregion that will reduce injury–related morbidity and mortality.

## Patients and Methods

### Study design and setting

This was a retrospective study of injured patients who presented to the accident and emergency (A&E) unit of University of Nigeria Teaching Hospital (UNTH) Ituku–Ozalla, in Enugu Southeast Nigeria, from January to December 2013. The UNTH is a 500–bed tertiary hospital that is located along the Enugu–Port Harcourt expressway.

The hospital provides trauma care to the residents of Enugu State and neighboring states of south–east and south–south Nigeria. It serves a population of about 8 million people. The research and ethics committee of UNTH Ituku–Ozalla approved the study protocol.

### Study population and data collection

All the patients seen with injuries at the A&E unit of the hospital from January to December 2013 were identified from the attendance register. The medical records of patients with complete documentations were reviewed. All patients with missing or incomplete medical records were not included in the study. Sociodemographic profile of the patients, etiology of the injury, injury–arrival interval, type of injury, injury severity score (ISS), comorbidities, duration of hospitalization, and outcome of treatment were extracted from the case files. Data extraction was done manually since there was no computerized trauma registry and entered into a data collection form designed for the study.

### Statistical analysis

Analysis of data was done using SPSS version 22.0 (SPSS Inc. Chicago, IL, USA). The frequencies of variables were identified using descriptive statistics. Chi–square test was used to analyze the data. Comparison of means was done using independent *t*–test. Statistical significance was set at *P* < 0.05.

## Results

A total of 1516 injured patients presented at the A&E unit during the study period. However, 789 patients (52%) had complete medical records and were included in the study. During the study period, 6238 patients presented to the A&E unit with both medical and surgical emergencies. This gives an injury prevalence of 24.3%. The ages of the patients ranged from 6 months to 95 years with a mean of 33.8 ± 18.1 years. The modal age group was 31–40 years, and majority of the patients were between 21 and 40 years of age (*n* = 371, 47.1%). There were 593 male (75.1%) and 196 female (24.9%) patients, with a male:female ratio of 3:1. Table 1 shows the demographic characteristics of the patients.

**Figure 1:**
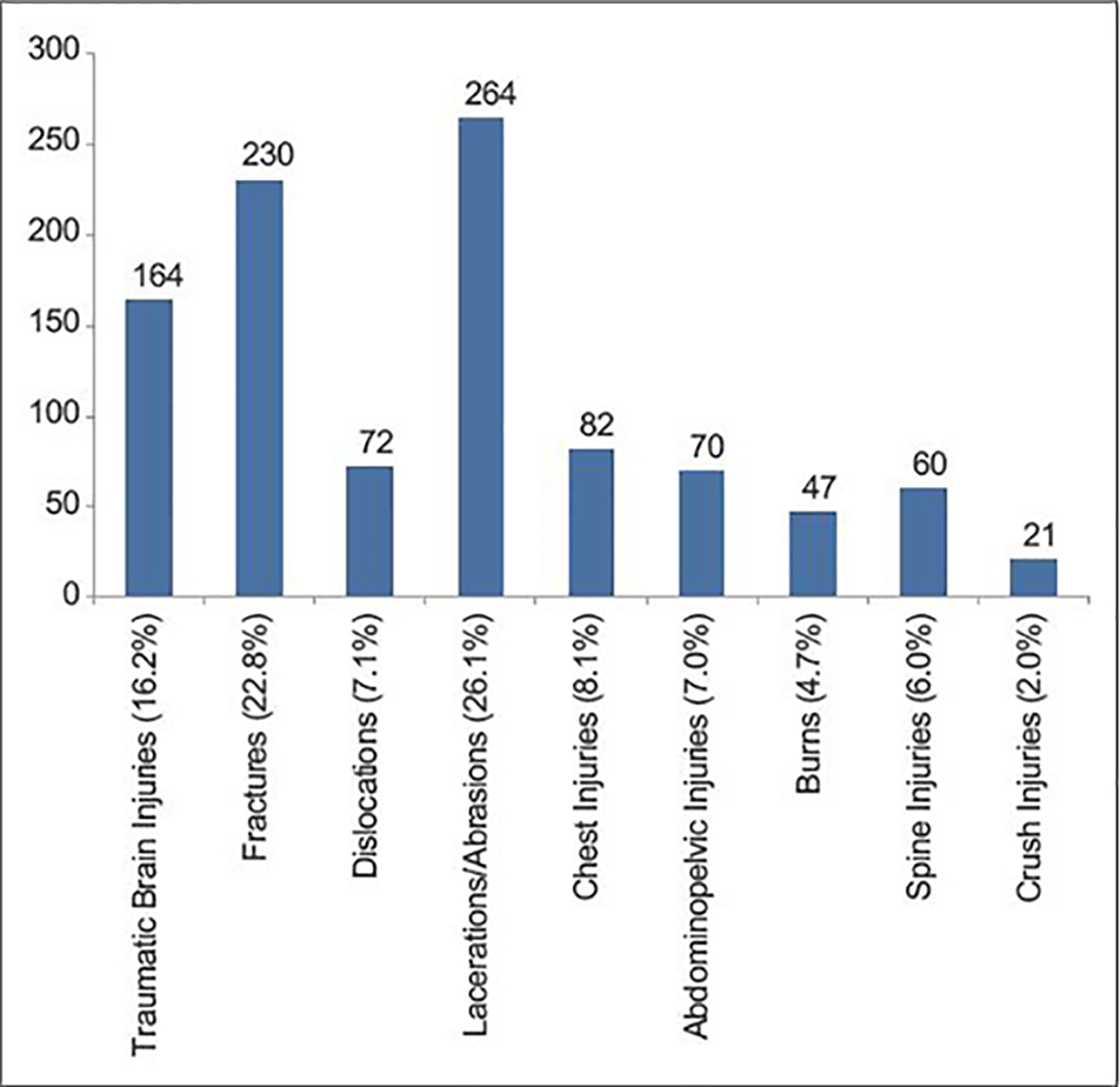
Distribution of injuries sustained by the patients

A total of 1010 injuries were recorded and 28% of the patients sustained multiple injuries. Analysis of the anatomical site of the injuries showed that the lower extremity was the most commonly injured body region (*n* = 200, 19.8%). This was closely followed by the head and face (*n* = 198, 19.6%) and the upper extremity (*n* = 143, 14.2%). The least injured body part was the perineum and external genitalia (*n* = 37, 3.7%). Figure 1 shows the distribution of injuries sustained by the patients.

Table 2 outlines the etiology of injuries. Analysis of injuries caused by RTA shows that motorcycle was the most common means of transportation (*n* = 132, 31%). This was followed by commuter buses (*n* = 116, 27.5%) and cars (*n* = 88, 21%). Pedestrian accident occurred in 45 (10.8%) cases of RTA while tricycles and lorries accounted for 25 (5.9%) and 16 (3.8%) of the RTAs, respectively.

There was no significant gender or occupational predilection in the etiology of the injuries (*P* = 0.11). However, there was a significant correlation between the age of the patients and the etiology of injuries (*P* = 0.0001) as shown in Table 3. Elderly patients and infants suffered mostly from falls; young adults suffered from RTA, gunshot injuries, assaults, and occupational injuries.

Analysis of injury characteristics shows that the tibia was the most common site of fracture (*n* = 55, 23.9%). Other sites of fracture were femur (*n* = 43, 18.9%), humerus (*n* = 38, 16.7%), radius/ulna (*n* = 33, 14.4%), and ankle (*n* = 30, 12.8%). Majority of the fractures were closed fractures (*n* = 161, 70%), 59 (25.6%) were open, and 10 (4.4%) were not stated. The tibia was the most common site of open fractures (*n* = 31, 52.2%). Majority of the open fractures were associated with motorbike accidents (*P* = 0.001).

A total of 164 (16.2%) patients sustained traumatic brain injury (TBI), out of which 52 (32%) had a Glasgow coma score (GCS) of 13–15 (mild); 62 (37.7%) were moderate (GCS: 9–12), and 50 (30.3%) were severe (GCS: 3–8). Spine injuries were recorded in 60 (7.6%) patients. Thirty-three (55.1%) occurred in the cervical spine, 25 (42.0%) occurred in the thoracic spine, while 2 (2.9%) occurred in the lumber region. Fifty–two (87.0%) of the spinal injuries were complete, while 8 (13%) were incomplete injuries.

The ISS of the patients ranged from 1 to 50 with a mean score of 8.9 ± 3.5. The mean ISS of male patients was 11.0 ± 4.1 and the mean ISS of the female patients was 7.6 ± 3.0. The gender difference in mean ISS was significant (*P* = 0.02). Table 4 shows the distribution of the ISS of the patients. Major trauma was defined as the ISS being >15. RTAs were responsible for 90.8% of patients with ISS >15. Patients with ISS >15 contributed to 64.1% of all deaths. Severe injuries were associated with early presentation to hospital and prolonged hospitalization (*P* = 0.001). Residual permanent disabilities were common among patients with ISS >15 (*P* = 0.03).

The injury–arrival interval ranged from 30 min to 4 weeks with a mean of 1.8 ± 1.1 days. Only 32 (4.1%) patients presented within the “golden hour” and 122 (15.5%) presented within 6 h. Majority of the patients (*n* = 214, 27.1%) presented within 24 h, while 111 (14.1%) presented after 7 days.

Duration of hospitalization ranged from 1 to 230 days with a mean duration of 12.7 ± 6.9 days. Majority of the patients (*n* = 529, 67.0%) were treated and discharged without permanent disability. Eighty–four (10.6%) were discharged with permanent disability (paraplegia, quadriplegia, and loss of body parts); 50 (6.3%) left against medical advice while 58 (7.4%) were referred to other hospitals. Sixty–eight patients died giving a mortality rate of 4.5%. Table 5 shows the distribution of mortality by etiology and type of injury.

## Discussion

The injury prevalence rate in our study was 24.3%. This suggests that about one in every four admissions into the emergency room was due to injury. This is similar to reports by Prekker *et al*.^[9]^ who reported 24.4% in Minnesota, USA. Another report by Adoga and Ozoilo^[10]^ in Jos, Nigeria reported a higher value of 31.1%. Frequent ethnic–religious crises in this region may account for this higher prevalence rate.

We noted a male preponderance in this study, and the mean age was similar to previous reports.^[11,12]^ Our study corroborates findings from other studies that the male young adults are most commonly involved in trauma.^[2,11,12]^ This is the active and productive age group of the society and results in huge economic losses.

RTA was the most common cause of injury in our study. The high prevalence of RTA has been reported by other authors in various countries.^[11–14]^ Motorcycle was the most common means of transportation among the victims of RTA. It is a popular means of commercial transportation in Nigeria and contributes significantly to the overall incidence of RTIs in developing countries.^[15]^ Therefore, provision of safer means of transportation and measures aimed at controlling motorcycle accidents will help to reduce the rising RTI epidemic in developing countries.

Fall was the second leading cause of injury in our study with a prevalence of 16%. Other studies in Nigeria by Elachi *et al*.^[11]^ and Thanni and Kehinde^[12]^ reported low prevalence of fall; 2.6% and 1.9%, respectively. In contrast, Huda *et al*.^[16]^ in India reported a fall prevalence of 29.5%. The higher incidence of fall in our study relative to the other Nigerian studies may be due to proximity of our hospital to rural communities where children and adolescents climb fruit trees in search of fruits. Domestic falls among elderly people in the rural communities may also be contributory. Fall from height was the most common mechanism of injury in children and adolescents while same level falls either during walking or standing was predominantly noted among the elderly. Similar pattern of falls has been reported by Wui *et al*.^[17]^ and Sterling *et al*.^[18]^

Soft–tissue injuries such as lacerations, avulsions, and abrasions were the most common injuries in our study. This was followed by fractures. This is consistent with reports from other Nigerian studies although the prevalences vary.^[11,12,19]^ The tibia and fibula were the most common site of fractures in our study. This was similar to results from other studies.^[15]^ Majority of the fractures were closed. Open fractures predominantly occurred in the tibia and were mostly related to motorcycle accidents (*P* = 0.001). This correlated with reports by Elachi *et al*.^[20]^

The lower limbs were the most common anatomical site of injury. This pattern was similar to the study by Thanni and Kehinde^[12]^ In contrast, Adoga and Ozoilo^[10]^ and Wui *et al*.^[17]^ reported the head as the most common site of injury in their study. The etiology and mechanisms of injury in the various studies may explain the difference.

The male patients had a higher mean ISS compared to the female patients. This suggests that males sustain more severe injuries than females. We noted that increased ISS up to a score of 25 was associated with increased length of hospitalization. However, beyond this score, the length of hospitalization decreased since the extent of injuries resulted in early death. Similar observations were reported by Yadollahi *et al*.^[21]^ Our study showed that patients with more severe injuries presented relatively earlier to hospital. The extent of injuries among these patients prompted the desire to seek early medical attention. This is in contrast to patients with less severe injuries who initially sought unorthodox care but later presented to hospital following development of complications or dissatisfaction. ISS has been reported to predict outcome and mortality in trauma patients.^[22,23]^ Our study corroborates these findings because ISS >15 was associated with increased mortality rate and residual permanent disabilities.

The mean injury–arrival time in our study was 1.8 days. This is very long when compared to 37.2 min reported by Bigdeli *et al*.^[24]^ in Iran and 36.3 min by Newgard *et al*.^[25]^ in a North American cohort study. This finding is an indicator of poor prehospital services in our environment. There is lack of ambulance services in most cities in Nigeria. Consequently, road crash victims are transported to hospitals by untrained sympathizers and passersby and this may have contributed to the long injury–arrival interval in our study. The widespread patronage of unorthodox and traditional healers by injured victims in our environment may also explain the delay in presentation to hospital.

Mortality in this report was 4.5% which was within the worldwide range of 0.5%–6%. This is comparable to 2% and 4.4% reported by Thanni and Kehinde^[12]^ and Solagberu *et al*.,^[19]^ respectively, in Nigeria and 4.7% by Egol *et al*.^[26]^ in USA. TBI was the most common cause of death and this is similar to reports from other studies.^[6,12,19,27]^ RTAs were responsible for majority of the deaths. This observation supports reports by other authors.^[6,7,12,19]^ Our study also noted that the most vulnerable road users were motorcyclists. Mortality was also associated with increase in ISS. Therefore, measures that aim to reduce the severity of injuries such as use of crash helmets and seat belts as well as improving the quality of emergency neurosurgical services in our subregion may help to improve the outcome of injuries.

This study also highlights the challenges of trauma research in developing countries like Nigeria, where national trauma databases are lacking. Only 52% of injured patients had complete medical records. The records were either missing or incomplete, making data retrieval and analysis difficult. There is need for establishment of computerized trauma registries in all tertiary health institutions in Nigeria, to ensure availability of complete patient medical records for audit.

## Conclusion

The injury prevalence in our study was 24.3%. The male young adults are predominantly involved in trauma with RTAs as the leading etiological factor. There was a significant correlation between etiology and the age of the patients. While the children and the elderly suffered from falls, young adults suffered from RTA, gunshot injuries, assaults, and occupational injuries. Soft–tissue injuries such as lacerations/abrasions and fractures were the most common type of injuries. Increased ISS was associated with early presentation to hospital, prolonged hospitalization, increased rate of permanent disabilities, and increased mortality rate. The trauma–related mortality rate was 4.5% with TBI as the leading cause of death.

### Recommendations

Massive public education and road safety campaigns will increase awareness and adherence to traffic safety regulations by road users and hence reduce the frequency of road crashes. The enforcement of road safety laws such as use of seat belt and crash helmets will significantly reduce the severity and fatality of injuries from RTAs. The construction and use of pedestrian bridges across very busy roads will reduce the frequency and severity of pedestrian injuries. Gunshot and assault–related injuries can be minimized by effective anti–crime and firearm control measures by governmental agencies.

## Figures and Tables

**Table 1:** Demographic characteristics of the patients (*n*=789)

	*n* (%)
Age (years)	
<1	17 (2.2)
1–10	87 (11.0)
11–20	72 (9.1)
21–30	170 (21.6)
31–40	201 (25.5)
41–50	107 (13.6)
51–60	57 (7.2)
61–70	38 (4.8)
71–80	31 (3.9)
>80	9 (1.1)
Sex	
Male	593 (75.1)
Female	196 (24.9)
Occupation	
Child/pupil/student	226 (28.6)
Driver/cyclist	68 (8.6)
Farmer	51 (6.5)
Civil servant/private employee	73 (9.3)
Unemployed	48 (6.1)
Businessman/trader	148 (18.8)
Artisan	115 (14.5)
Others	60 (7.6)
Marital status	
Single	401 (50.8)
Married	348 (44.1)
Widow	34 (4.3)
Separated	4 (0.5)
Divorced	2 (0.3)
Level of education	
Primary	132 (16.7)
Secondary	227 (28.8)
Tertiary	128 (16.2)
No Education	51 (6.5)
Not Stated	251 (31.8)

**Table 2:** Etiology of injuries

Etiology	*n* (%)
RTA	421 (53.3)
Fall	126 (16.0)
Gunshot injury	79 (10.0)
Burns	31 (3.9)
Sports injury	22 (2.8)
Industrial injury	38 (4.8)
Assault	60 (7.6)
Birth trauma	12 (1.5)
Total	789 (100)

RTA=Road traffic accident

**Table 3:** Distribution of etiology of injuries by age

Age group (years)	Etiology								
	RTA	Fall	Gunshot	Burns	Sports injury	Industrial injury	Assault	Birth trauma	Total
<1	3	2	-	-	-	-	-	12	17
1–10	31	35	-	7	10	-	4	-	87
11–20	25	10	3	5	9	4	16	-	72
21–30	108	10	23	3	-	10	16	-	170
31–40	127	8	28	7	3	12	16	-	201
41–50	59	11	16	7	-	8	6	-	107
51–60	40	6	7	2	-	2	-	-	57
61–70	16	16	2	-	-	2	2	-	38
71–80	12	19	-	-	-	-	-	-	31
>80	-	9	-	-	-	-	-	-	9
Total (%)	421 (53.3)	126 (16.0)	79 (10.0)	31 (3.9)	22 (2.8)	38 (4.8)	60 (7.6)	12 (1.5)	789 (100)

Pearson’s *χ*^2^=505.6, *P*=0.0001. RTA=Road traffic accident

**Table 4:** Injury severity score of the patients

Injury severity score	*n* (%)
1–8 (minor)	351 (44.5)
9–15 (moderate)	272 (34.4)
16–24 (serious)	92 (11.7)
25–49 (severe)	70 (8.9)
50–74 (critical)	4 (0.5)
75 (maximum)	0 (0)
Total	789 (100)

**Table 5:** Distribution of mortality by etiology and type of injury

Etiology	Type of injury						
	TBI	Spine injury	Burns	Abdominopelvic Injury	Chest injury	Multiple injury	Total (%)
RTA	20	8	-	6	2	9	45 (66.1)
Gunshot injury	2	-	-	4	4	-	10 (14.7)
Assault	1	-	-	-	-	2	3 (4.4)
Burns	-	-	2	-	-	-	2 (3.0)
Fall	2	4	-	-	-	-	6 (8.8)
Occupational injury	2	-	-	-	-	-	2 (3.0)
Total (%)	27 (39.7)	12 (17.6)	2 (2.9)	10 (14.7)	6 (8.8)	11 (16.2)	68 (100)

Person’s *χ*^2^=64.3, *P*=0.001. TBI=Traumatic brain injury; RTA=Road traffic accident
